# Ischiofemoral impingement: the evolutionary cost of pelvic obstetric adaptation

**DOI:** 10.1093/jhps/hnab004

**Published:** 2021-02-08

**Authors:** E A Audenaert, K Duquesne, J De Roeck, T Mutsvangwa, B Borotikar, V Khanduja, P Claes

**Affiliations:** 1 Department of Orthopedic Surgery and Traumatology, Ghent University Hospital, Corneel Heymanslaan 10, Ghent 9000, Belgium; 2 Department of Trauma and Orthopedics, Addenbrooke's Hospital, Cambridge University Hospitals NHS Foundation Trust, Hills Road, Cambridge CB2 0QQ, UK; 3 Department of Electromechanics, Op3Mech Research Group, University of Antwerp, Groenenborgerlaan 171, Antwerp 2020, Belgium; 4 Department of Human Structure and Repair, Ghent University, Corneel Heymanslaan 10, Ghent 9000, Belgium; 5 Division of Biomedical Engineering, University of Cape Town, Anzio Rd, Observatory, Cape Town 7925, South Africa; 6 Symbiosis Center for Medical Image Analysis, Symbiosis International University, Lavale, Mulshi District, Pune 412115, India; 7 Laboratory of Medical Information Processing (LaTIM), UMR 1101, INSERM, Avenue Foch 12, 29200 Brest, France; 8 Department of Human Genetics, KU Leuven, Herestraat 49, 3000 Leuven, Belgium; 9 Medical Imaging Research Center, University Hospitals Leuven, Herestraat 49, 3000 Leuven, Belgium; 10 Department of Electrical Engineering, ESAT/PSI, KU Leuven, Herestraat 49, 3000 Leuven, Belgium; 11 Murdoch Children’s Research Institute, Melbourne, Flemington Road, Parkville Victoria 3052, Australia

## Abstract

The risk for ischiofemoral impingement has been mainly related to a reduced ischiofemoral distance and morphological variance of the femur. From an evolutionary perspective, however, there are strong arguments that the condition may also be related to sexual dimorphism of the pelvis. We, therefore, investigated the impact of gender-specific differences in anatomy of the ischiofemoral space on the ischiofemoral clearance, during static and dynamic conditions*.* A random sampling Monte-Carlo experiment was performed to investigate ischiofemoral clearance during stance and gait in a large (*n* = 40 000) virtual study population, while using gender-specific kinematics. Subsequently, a validated gender-specific geometric morphometric analysis of the hip was performed and correlations between overall hip morphology (statistical shape analysis) and standard discrete measures (conventional metric approach) with the ischiofemoral distance were evaluated. The available ischiofemoral space is indeed highly sexually dimorphic and related primarily to differences in the pelvic anatomy. The mean ischiofemoral distance was 22.2 ± 4.3 mm in the females and 29.1 ± 4.1 mm in the males and this difference was statistically significant (*P* < 0.001). Additionally, the ischiofemoral distance was observed to be a dynamic measure, and smallest during femoral extension, and this in turn explains the clinical sign of pain in extension during long stride walking. In conclusion, the presence of a reduced ischiofemroal distance and related risk to develop a clinical syndrome of ischiofemoral impingement is strongly dominated by evolutionary effects in sexual dimorphism of the pelvis. This should be considered when female patients present with posterior thigh/buttock pain, particularly if worsened by extension. Controlled laboratory study.

## INTRODUCTION 

**Fig. 1.**
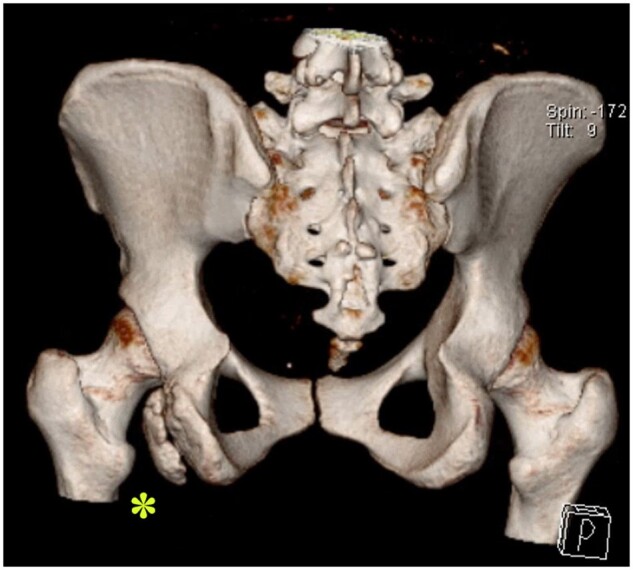
Secundary ischiofemoral impingement in a 20-year-old gymnast following apophysiolysis of the left tuber ischiadiucum (asterisk).

Ischiofemoral impingement is one of the extra-articular hip impingement syndromes which is a potential cause of posterior hip pain [1]. The basic pathology is that the space between the lesser trochanter on the femur and the ischial tuberosity on the pelvis, known as the ischiofemoral space, is reduced and this leads to compression of the quadratus femoris muscle within the space causing pain [2, 3]. A decreased ischiofemoral distance has been consistently identified in the radiographic assessment of patients diagnosed with the condition. While the vast majority of cases are idiopathic, non-idiopathic causes have been reported as secondary femoral pathology, e.g. lesser trochanteric fracture or avulsion or following valgus osteotomy of the hip or pelvic pathology, e.g. ischial tuberosity apophysitis or avulsion in young athletes (Fig. 1) [4]. Interestingly, there appears to be a strong female predominance [2, 5, 6].

The human pelvis is well known to be highly sexual dimorph. This is mainly due to the evolutionary obstetric adaptation of the female pelvis [7–10]. This profound dimorphism of the pelvis is intrinsically linked to the ‘obstetric dilemma’, the evolutionary trade-off in females between two competing demands: biomechanical efficiency versus reproduction. A wide pelvis allows for the birth of large brained infants, however, it also results in decreased hip abductor efficiency for pelvic stabilization during the single-leg support phase of walking and running. Nature has found a compromise mainly in the following two adaptations. Firstly, there is a larger pelvic width for height ratio in females [11]. In absolute values, the pelvic width is only slightly larger in females as compared to males [12–14]. However, males present with a larger body size by sexual selection and demonstrate a larger femoral offset. This difference in pelvic width for body size, equalizes locomotor costs between males and females [15, 16].

A second evolutionary adaption of the female pelvis involves hemipelvic rotational changes, enlarging the pelvic canal (obstetric selection), thereby resulting in an increased intertuber diameter and interspinous distance as well as the acetabular version, while maintaining a nearly equal interacetabular distance as compared to males [12, 13, 17, 18]. This hemipelvic version, however, affects the position of the entire posterior pelvic column in its relation to the femur [19]. Combining these adaptation changes with a smaller overall height in females and therefore a smaller femoral offset, the available space between the ischium and the femur is significantly reduced in females, therefore, increasing a sex-specific risk for ischiofemoral impingement.

**Fig. 2.**
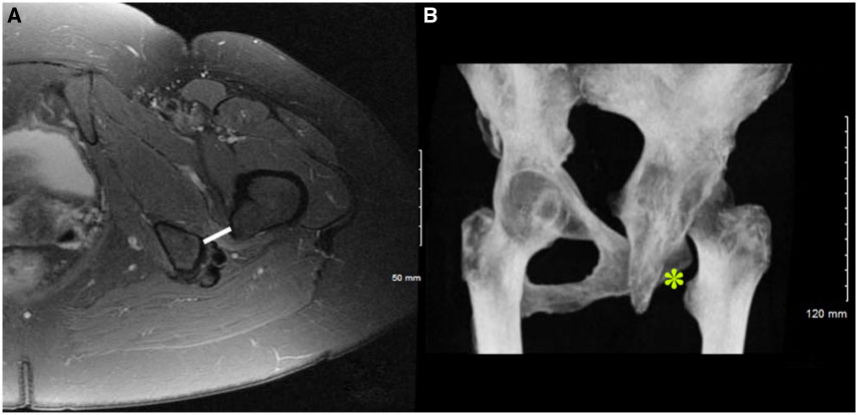
Radiographic assessment in a patient suspected with ischiofemoral impingement, demonstrating a narrow ischiofemoral distance on MRI (**A**) and 3D reconstructions (**B**) in combination with edema of the quadratus femoris muscle (A).

Clinically, prolonged irritation of squeezed structures presents as lower buttock and groin pain which can be elicited during long stride walking. Patients will demonstrate posterior hip pain or deep medial groin pain, which can be provoked with the ischiofemoral impingement test: extension, external rotation and adduction of the hip [20]. Radiographically, a narrow ischiofemoral space on AP pelvis and axial slices of computed tomography (CT) and magnetic resonance imaging (MRI) can be observed [21]. Additionally, signal changes ranging from edema to atrophy and/or fatty degeneration of the quadratus femoris muscle and surrounding structures can be seen on an MRI scan in long standing cases (Fig. 2) [2, 6, 22, 23].

The diagnostic and therapeutic challenges of ischiofemoral impingement are significant. Ischiofemoral space is a dynamic parameter and recent studies have proposed MRI scans through full range of hip motion [24, 25]. However, scanning techniques are still limited to non-weight bearing passive motion and thus may lead to a missed diagnosis. Furthermore, there is an inflammatory component which responds to injections of local anesthetic and steroids and some cases are amendable to physiotherapy [2, 5]. A recent systematic review on the treatment strategies for ischiofemoral impingement revealed that several treatment strategies had been reported for ischiofemoral impingement, and most of them had good short- to medium-term outcomes with a low rate of complications. However, there were no comparative studies to assess the superiority of one technique over another, thus further research was recommended [26, 27]. Hence, due to the limited understanding of the condition, preventive action in sports and the mechanism behind success of conservative treatment remains unelicited. Furthermore, the literature has mainly focused on the risk factors for developing ischiofemoral impingement on the femoral side, whereas the pelvic morphology has grossly remained uninvestigated, probably due to its complex morphology [21, 28, 29]. Nevertheless, from an evolutionary point of view, the contribution of the pelvis is likely to be of significant importance.

With recent advances in computer graphics and vision, data-driven techniques allow us to explore a more grounded description of anatomical variation in a population using large samples of input data [30]. Using geometric morphometric methods, it is possible to accurately describe the anatomy and its variation for any population using conventional multivariate statistics from sets of homologous landmarks representing the shape of the underlying structures. Unlike existing approaches, these techniques provide accurate parameterizations of an individual’s morphology and describe the anatomical distribution of an entire population. Moreover, these models are useful in improving the diagnosis, classification and treatment of specific medical conditions [31].

The aim of our study, therefore, was to improve the understanding of causation of ischiofemoral impingement, by investigating the gender-specific impact of anatomical variation on the ischiofemoral space, both in static dynamic conditions and based on a validated geometric morphometric approach.

## MATERIALS AND METHODS

### Patient population and pseudo-kinematic protocol

The Ghent Lower Limb model was used to generate a virtual cohort of 20 000 male and an equal number of female cases, representative of a western European population. The model is one of the most comprehensive morphometric lower limb models in the literature and has been validated in terms of sexual specificity, population coverage, model specificity, generalizability and accuracy. It is composed by performing principal component analysis (PCA) of dense corresponding surface meshes obtained from 544 lower limb segmentations (362 male and 182 female training samples with an average age of 67.8 ± 10.8 and 69 ±13.3 years, respectively). The model was cleared from any positional variance (noise) that occurred at the time of image acquisition, therefore providing absolute standardization of hip positioning prior to morphometric analysis [32–36]. The imaging database on which the model is based was constructed from living subjects receiving angio-CT scanning for vascular work-out between 2012 and 2016. The participating subjects were not exposed to additional radiation for the present study. The assembly of the imaging data base and its use was reviewed and approved by the ethics committee of the Ghent University Hospital (under reference B670201111480) The study protocol conforms to the ethical guidelines of the 1975 Declaration of Helsinki and was performed according to institutional guidelines. Informed consent was obtained from all subjects. No subjects are under 18 years of age were included.

After segmentation, registration and pose denoising of the lower limb anatomy, male and female shape entries are separated and used for the construction of two distinct point distribution models to describe the sexually dimorphic shape variance Mathematically, each model was described using the following generative equation for a point distribution model:

$$S=\overset{-}{S}+Pb$$

where *S* is a vector of size three times n representing the shape in terms of *n* number of three dimensional (3D) landmark points. In the equation above $\overset{-}{S}\;$corresponds to the average shape of the model and *P* represents a *t* × 3*n* matrix encapsulating *t* eigenvectors describing the principal directions of variation of the model. Furthermore, every unit eigenvector, referred to as shape mode, is associated with an eigenvalue λ_i_, *i* ∈ {1, ⋯, *t*} which describes the magnitude of variation along each axis. Lastly, *b* = (*b*_1_, ⋯, *b*_t_) represents a vector containing the b_i_ weights that regulate the deviation of the shape *S* from the mean and follow a normal distribution.

It was previously determined that 20 shape modes would largely sufficed to describe a sizable 99% of the total anatomical variance for both sexes [32]. We, therefore, retained *t* = 20 modes of variation to generate the virtual shapes, by randomly varying the normal distributed deviation weights b_i_ within each point distribution model (Monte Carlo sampling). Doing so, a virtual population of 20 000 cases per sex class was generated and used to evaluate the ischiofemoral distance in stance and during gait.

The hip joint was modeled as a spherical joint with three degrees of rotational freedom. Gender-specific hip rotations during walking were obtained from the documented cases of the Orthoload Database and were subsequently imposed on all 40 000 models [37]. Simulations were performed on a Dell EMC PowerEdge 940 server hosting 72 cores, processing in parallel at 2.9 GHz.

### Anatomical descriptors

**Fig. 3.**
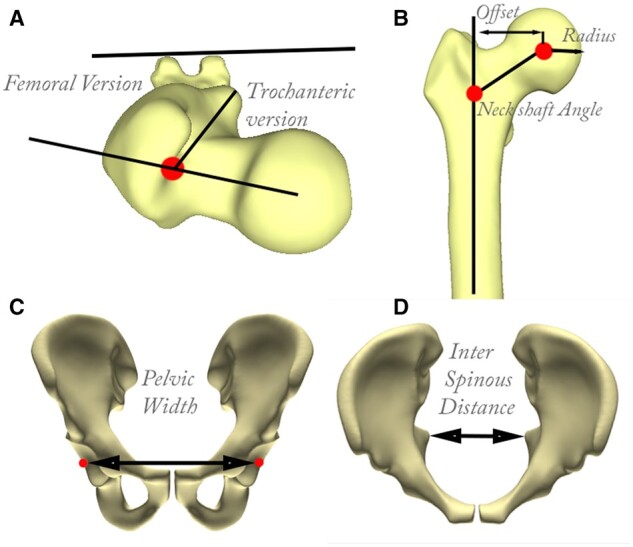
Anatomical descriptors used to evaluate the impact of ischiofemoral distance on gender-specific gait and shape. (**A** and **B)** The femoral anatomic measures of femoral anteversion, lesser trochanter retroversion, femoral offset, femoral neck–shaft angle and head radius for the femur. (**C** and **D)** pelvic anatomic measures of pelvic width and interspinous distance shown in the frontal plane.

Well established sagittal and axial measures were obtained in both male and female virtual populations to describe variance and to relate with a decreased ischiofemoral distance. These measures include femoral anteversion, lesser trochanter retroversion, femoral offset, femoral neck–shaft angle and radius of the femoral head (Fig. 3). On the pelvis, the pelvic width and interspinous distance were evaluated. Hemipelvic rotation was discretized by the ratio of the interspinous distance and pelvic width. Landmarks to determine these measures were established on the gender-specific mean model shapes and were automatically transferred during the virtual population generation step. On each virtual model, the ischiofemoral distance was defined as the shortest distance between the lessor trochanter area and the pelvic bone by using a nearest neighbor search in Matlab ^®^ (R2019b, Mathworks Inc., Natick, MA, USA).

### Statistical analysis

Given the profound dominance of size in shape models of human anatomy in the Euclidian subspace [38], the ischiofemoral distance-shape relationship was evaluated by means of canonical correlation analysis (CCA) in the Mahalanobis subspace. In particular, the principal component values of the shape data, serving as predictor variables for the observed ischiofemoral distance measures as response variables, were used. Overall explained variance in the observed shape components by the ischiofemoral distance measurement was evaluated using partial least squares regression (PLSR). CCA was used to report on the statistical correlation, while PLSR was used to define the predictive value of the ischiofemoral distance feature. PLSR was further used to describe the male and female consensus shape configurations correlating with low and excess ischiofemoral distance.

## RESULTS

### Sexual dimorphism in pelvifemoral anatomy

**Fig. 4.**
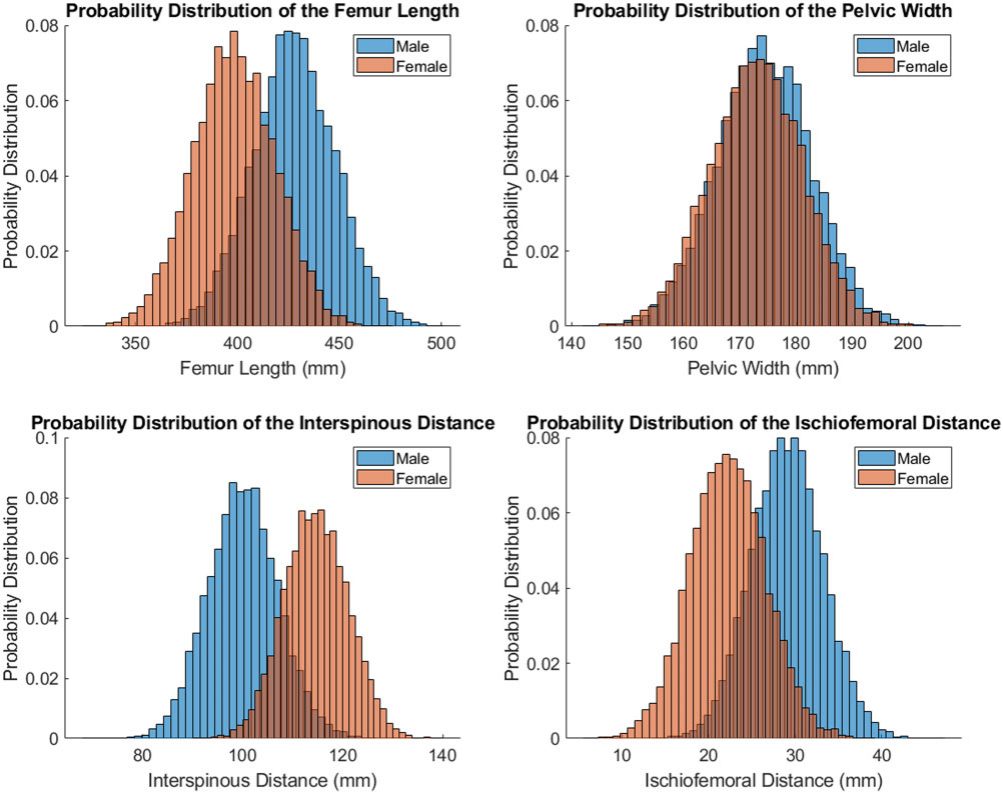
Pelvifemoral anatomic differences between sexes in the virtual population.

The femoral length was used to evaluate differences in body size, whereas pelvic variation was evaluated by means of the pelvic width and interspinous distance. While the pelvic width did not differ largely between male and female, the male femur length, offset and femoral head radius were significantly larger (Fig. 4), whereas the interspinous distance followed the inverse relationship. Details of all variables evaluated can be found in Table I.

**Table I.** Table showing mean values and standard deviations of common used discrete measures to describe femur and pelvic morphology and sex differences

	Male (*n* = 20 000)	Female (*n* = 20 000)	*P* value
Neck–shaft angle (°)	125.22 ± 5.48	126.44 ± 5.51	<0.001
Femoral anteversion (°)	9.86 ± 7.07	10.82 ± 6.06	<0.001
Femoral offset (mm)	38.05 ± 7.72	33.21 ± 6.80	<0.001
Ischiofemoral distance (mm)	29.09 ± 4.12	22.24 ± 4.32	<0.001
Head radius (mm)	25.29 ± 1.06	22.37 ± 1.02	<0.001
Lesser trochanter retroversion (°)	27.17 ± 7.70	27.23 ± 8.31	<0.001
Femoral length	427.68 ± 20.12	398.24 ± 19.99	<0.001
Pelvic width	174.30±8.36	172.92 ± 8.23	<0.001
Ischial spine width	100.04 ± 7.01	114.68 ± 6.41	<0.001
Ratio pelvic/ischial spine width	0.57 ±0.04	0.66 ±0.03	<0.001

### Anatomical predictors of a decreased ischiofemoral distance

Weak to moderate correlations with the ischiofemoral distance could be found for the femoral offset and to a lower extent the lesser trochanter version. Moderate to strong correlations were observed for the interspinous and, in particular, the ratio describing the interspinous distance, normalized for the pelvic width. There was a very strong correlation with the overall hip geometry and the ischiofemoral distance, explaining 7.1% of the variance for male anatomy and 7.8% of the variance for female anatomy (*P* values < 0.001) (Table II).

**Table II.** List of the femur morphological parameters correlations with the minimal ischiofemoral distance

Correlations with the ischiofemoral distance	Correlation coefficient *r*	*P* value	Correlation coefficient *r*	*P* value
	Male (*n* = 20 000)		Female (*n* = 20 000)	
Neck–shaft angle (°)	**−**0.38	<0.001	**−**0.37	<0.001
Femoral anteversion (°)	**−**0.31	<0.001	**−**0.29	<0.001
Femoral offset (mm)	**0.46**	<0.001	**0.40**	<0.001
Ischiofemoral distance (mm)	1	<0.001	1	<0.001
Head radius (mm)	0.17	<0.001	0.24	<0.001
Lesser trochanter retroversion (°)	**0.43**	<0.001	0.34	<0.001
Femoral length	0.06	<0.001	0.12	<0.001
Pelvic width	0.15	<0.001	0.20	<0.001
Interspinous distance	**−**0.27	<0.001	**−0.54**	<0.001
Normalized interspinous distance	**−0.41**	<0.001	**−0.72**	<0.001
Hip shape (SSM, corrected for size)	**0.95**	<**0.001**	**0.96**	<**0.001**

SSM, statistical shape model. All significance values are in the column aside. The bold values are just the relevant & significant findings (*r* > 0.4 and *P* < 0.001) as indicated in the table.

### The ischiofemoral distance during gait

**Fig. 5.**
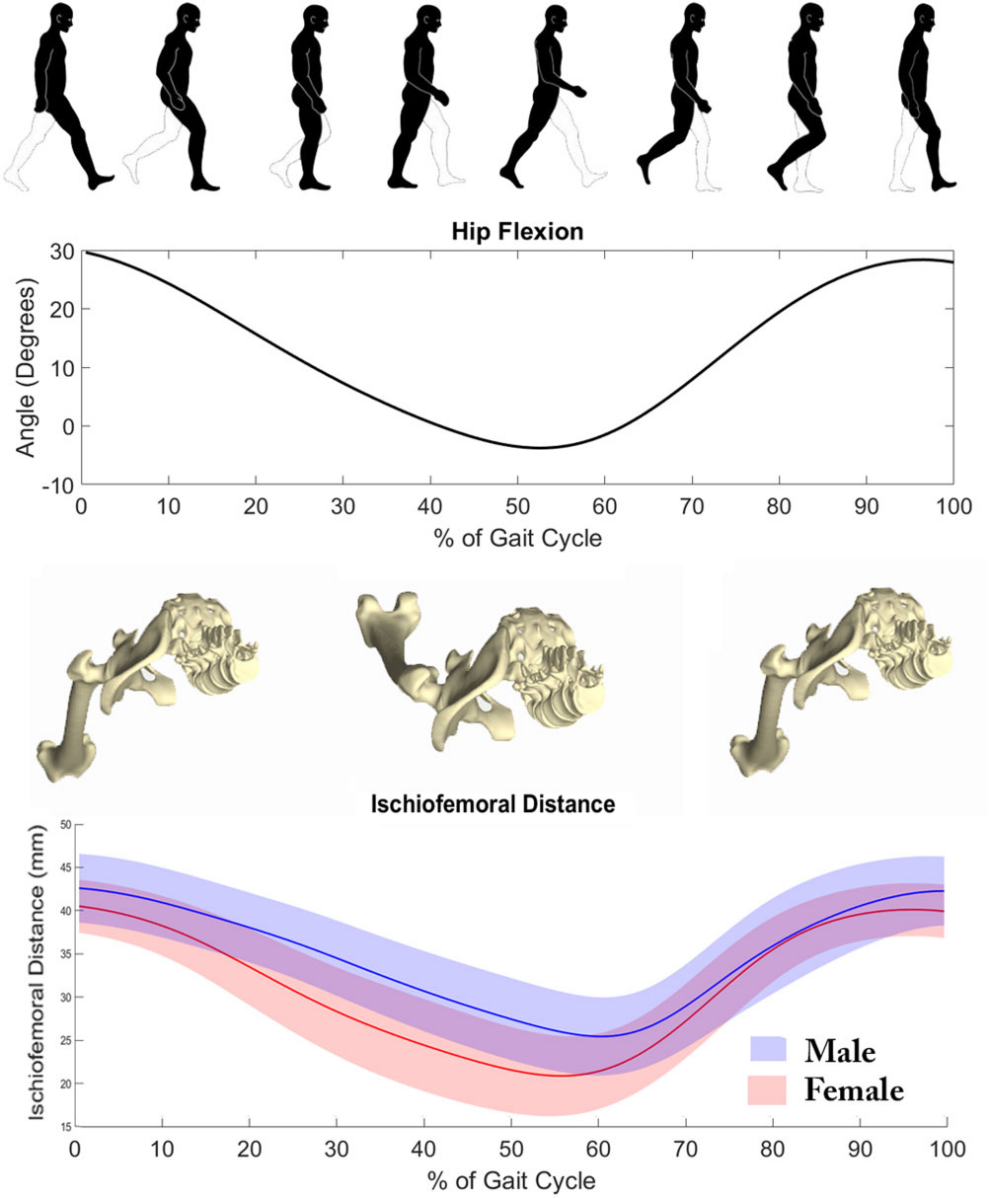
Ischiofemoral distance determined as a function of hip flexion angle on each of 40 000 virtual population models. Minimum ischiofemoral distance was observed during terminal stance to pre-swing phase of the gait cycle.

The ischiofemoral distance was further evaluated during gait, demonstrating a decrease in ischiofemoral distance values upon hip extension and an increase in ischiofemoral distance values during flexion (Fig. 5). Again, females presented with consistently smaller ischiofemoral spaces compared to males. In both genders, minimum ischiofemoral distance occurred during terminal stance to pre-swing phase of the gait cycle.

### Shape regressions using ischiofemoral distance values

**Fig. 6.**
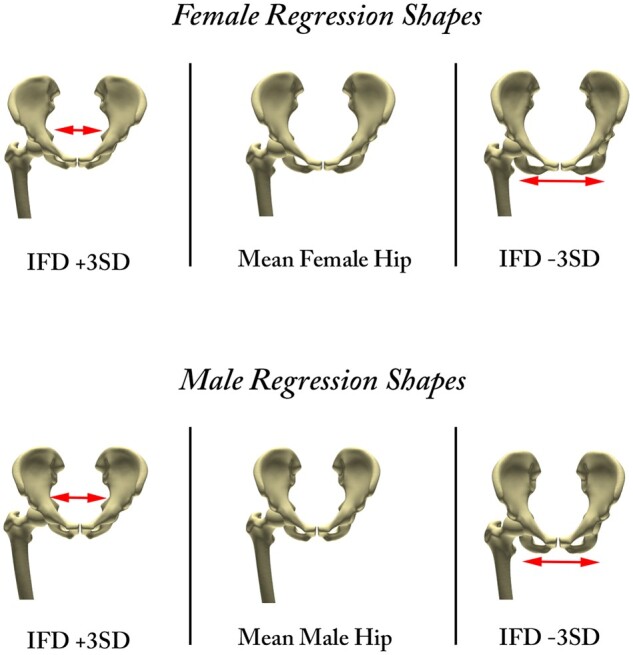
Gender-specific shape regressions for ±3 standard deviations of ischiofemoral distance calculated from the virtual population. The impact of hemipelvic version is shown by red arrows.

Finally, a regression analysis was performed to evaluate the correlation of hip geometry with increasing and decreasing values of ischiofemoral distance in both genders. Here, the impact of hemipelvic version, discretized by the interspinous distance, on the ischiofemoral distance was apparent in both sexes (Fig. 6).

## DISCUSSION

Statistical population modeling presents an exciting and novel means for non-invasive testing and the evaluation of physiology and biomechanical variability across populations [39–41]. The unparalleled advantage of population numbers in virtual population models allows for the identification and study outlier condition of function and shape, in cohort sizes beyond what is clinically achievable [42]. Based on the current study on a virtual population compromised of 40 000 cases, we were able to describe variation in ischiofemoral distance and identify anatomical risk descriptors for pelvic and femoral morphology. Two important observations were able to be made. Firstly ischiofemoral distance was indeed highly sexually dimorphic and primarily related to differences in pelvic anatomy. Secondly, ischiofemoral distance was a dynamic measure, and appeared at its smallest value during femoral extension, explaining the clinic sign of pain in extension during long stride walking.

### Pelvic anatomy

While discrete measures to depict femoral morphology are well established in the literature, they remain elusive for the pelvis due to its complex 3D morphology which is difficult to capture on 2D X-ray projections or 2D slices on CT and MRI imaging. This might explain the lack of studies particularly investigating the role of pelvic sexual dimorphism in young adult hip pathology. The obstetric specialization of the female pelvis is however well documented in numerous research areas, reflected in an unquestioned interest of the pelvis in metrics based sex determination, in forensic and evolutionary sciences. Studies of sexual dimorphism in the human skeleton show that while, in general, many characteristics reflect full body size, and are therefore larger in men than in women, the particular dimensions of the pelvic canal follow the inverse model [11, 43].

In the present study, a novel parameter, the ischial spine ratio was described. The ratio provides a measure for the interspinous diameter, normalized by the pelvic width. The interspinous diameter, although uncommonly used in sports and orthopedic literature, is a popular pelvimetric measure in the obstetric literature. The position of the ischial spine has been strongly correlated with the version of the acetabulum and the hemipelvis [19]. The ischial spine projection into the pelvis was introduced by Swiss Hip Group as an important sign of retroversion of the hemipelvis. Furthermore, the sign has been demonstrated to highly correlate with the well-known acetabular crossover sign [19]. Normalizing the interspinous diameter by the pelvis width provides a continuous measure for the ischial spine sign and therefore hemipelvic version. A decreased ischial spine ratio indicates hemiplevic retroversion and medialization of the ischial tuberosity and thereby an increased ischiofemoral distance, whereas an increase in the ischial spine ratio describes hemipelvic anteversion, lateralization if the ischial tuberosity and thereby a decreased ischiofemoral distance. By nature and obstetric evolution, female patients, for a similar pelvic width present with a larger interspinous diameter and as such increased acetabular anteversion as opposed to males. This means as well, that for a nearly equal pelvic width, females will have smaller ischiofemoral distances. Although not specifically investigated, clinical series on ischiofemoral impingement confirm this sex predominance with females being present in 83–100% of clinical series presented [2, 5, 6]. Furthermore, the observation of important sexual dimorphism in the ischial spine position challenges the value of the ischial spine projection as a unisex measure for acetabular anteversion.

### Dynamic nature of ischiofemoral impingement

A second important observation in the current study relates to dynamic changes in the ischiofemoral distance. As the ischial tuberosity is posteriorly positioned relative to the femur and the lesser trochanter, the distance reduces during extension and increases with flexion. This finding was also observed by Kivlan *et al*. [20] based on a cadaveric study. Extrapolating this finding means that the ischiofemoral distance will change with pelvic positional variation, namely increase with anterior pelvic tilt and decreased with a posterior pelvic tilt. Whilst authors have suggested static and dynamic pelvic tilt can be affected by training or become therapeutic in the cases of femoroacetabular impingement, it appears to have the opposite type of effect in patients with ischiofemoral impingement. Further clinical trials are clearly mandatory in this arena but from the data provided here, these studies can be designed and substantiated.

### Psoas snapping and ischiofemoral impingement

While without any doubt, the strongest correlation with a decreased ischiofemoral distance was observed in the pelvic morphology, a weaker but equally significant correlation with the femoral anatomy was present as well. Strength of these correlation and direction were in good agreement with earlier reports in the literature [21]. Interestingly, the ischiofemoral distance correlated with a number of anatomical measures that have been previously related to psoas snapping as well, in particular excess femoral anteversion, a decreased femoral offset and valgus [32, 44]. Audible snapping has indeed been reported as a clinical sign in ischiofemoral impingement cases [1, 2, 45]. While psoas snapping has been previously predominantly related to femoral anatomical variation, there appears to be a certain overlap with risk factors for ischiofemoral impingement and one should rule out ischiofemroal impingement, in any case, presenting with an audible psoas snap combined with deep gluteal pain [46].

### Limitations

The present work needs to be interpreted within is methodological limitations. Firstly, an important restriction of the presented work relates to the population model implemented, namely a training set of shape samples obtained from the Belgian population and the unknown extent of which findings can be extrapolated to other populations, especially those further distinct in genetic background. The complex interaction between genes, environment and culture, results in a population-based variation. Numerous studies have indeed demonstrated that the appropriate evaluation of this variation necessitates specific standards for each population [47, 48]. With respect to the relationship between pelvic dimorphism and body size in humans, higher pelvic dimorphism in smaller-bodied human populations generated by stronger obstetric selection has been described relative to larger-bodied populations [11]. Nevertheless, absolute findings and correlations of all measures mirror well findings in the literature [21, 49].

A second restriction relates to the age of the study population, which originates from adults all beyond 40 years of age. Human pelvic dimorphism has been found to follow a complex developmental trajectory. Both sexes follow very similar developmental trajectories until puberty where the female trajectory diverges substantially from the common course up to the age of 25–30 years. From 40 years onward females resume a mode of pelvic development similar to males, resulting in significant reduction of obstetric dimensions [50]. Such age-related morphology changes have indeed been observed by Khanduja *et al*. [21], who described a decreasing ischiofemoral distances with age. It can therefore be expected that the clinical presentation of ischiofemoral impingements is most pronounced in young adult females who on top of their developmental trajectory are much more active in sports and work-related activities at this point of their life.

In conclusion, the clinical syndrome of ischiofemoral impingement is characterized by dynamic metrics of the ischiofemoral space and is strongly dominated by evolutionary effects of sexual dimorphism on the pelvis. This observation urges for a strong revision of our current diagnostic and therapeutic view on the condition.

## FUNDING 

E.A. was supported a senior clinical research fellowship by the Flemmish research foundation (FWO). P.C. was supported by grants from the Research Fund KU Leuven (BOF-C1, C14/15/081), the Research Program of the Fund for Scientific Research—Flanders (Belgium; FWO, G078518N) and the US National Institutes of Health (1-RO1-DE027023).

## CONFLICT OF INTEREST STATEMENT

None declared. 

## PATIENT AND PUBLIC INVOLVEMENT

Patients and/or the public were not involved in the design, or conduct, or reporting, or dissemination plans of this research.

## PATIENT CONSENT FOR PUBLICATION

Informed consent was obtained from all subjects. No subjects are under 18 years of age were included.

## ETHICS APPROVAL

The imaging database on which the study was performed was constructed from living subjects receiving angio-CT scanning for vascular work-out between 2012 and 2016. The participating subjects were not exposed to additional radiation for the present study. The assembly of the imaging database and its use was reviewed and approved by the ethics committee of the Ghent University Hospital (under reference B670201111480). Informed consent was obtained from all subjects. No subjects are under 18 years of age were included.

## PROVENANCE AND PEER REVIEW

Not commissioned; externally peer reviewed.

## DATA AVAILABILITY

The datasets generated for this study are available on reasonable request to the corresponding author. The data can be obtained from the first author, E.A. There is no time limit on the availability of the data.
